# Activation and Expression of Peroxisome Proliferator-Activated Receptor Alpha Are Associated with Tumorigenesis in Colorectal Carcinoma

**DOI:** 10.1155/2019/7486727

**Published:** 2019-07-03

**Authors:** Tatsuya Morinishi, Yasunori Tokuhara, Hiroyuki Ohsaki, Emi Ibuki, Kyuichi Kadota, Eiichiro Hirakawa

**Affiliations:** ^1^Laboratory of Pathology, Department of Medical Technology, Kagawa Prefectural University of Health Sciences, Takamatsu, Kagawa 761-0123, Japan; ^2^Department of Medical Technology, Ehime Prefectural University of Health Sciences, Tobe, Ehime 791-2101, Japan; ^3^Laboratory of Pathology, Department of Medical Biophysics, Kobe University Graduate School of Health Sciences, Kobe, Hyogo 654-0142, Japan; ^4^Department of Diagnostic Pathology, University Hospital, Faculty of Medicine, Kagawa University, Miki, Kagawa 761-0793, Japan

## Abstract

Peroxisome proliferator-activated receptor alpha (PPAR-*α*) belongs to the PPAR family and plays a critical role in inhibiting cell proliferation and tumorigenesis in various tumors. However, the role of PPAR-*α* in colorectal tumorigenesis is unclear. In the present study, we found that fenofibrate, a PPAR-*α* agonist, significantly inhibited cell proliferation and induced apoptosis in colorectal carcinoma cells. In addition, PPAR-*α* was expressed in the nucleus of colorectal carcinoma cells, and the expression of nuclear PPAR-*α* increased in colorectal carcinoma tissue compared with that of normal epithelium tissue (P<0.01). The correlation between the expression of nuclear PPAR-α and clinicopathological factors was evaluated in human colorectal carcinoma tissues, and the nuclear expression of PPAR-*α* was significantly higher in well-to-moderately differentiated adenocarcinoma than in mucinous adenocarcinoma (P<0.05). These findings indicate that activation of PPAR-*α* may be involved in anticancer effects in colorectal carcinomas, and nuclear expression of PPAR-*α* may be a therapeutic target for colorectal adenocarcinoma treatment.

## 1. Introduction

More than 1.2 million colorectal carcinomas are diagnosed every year, accounting for approximately 10% of all carcinomas worldwide [1]. There was considerable progress in the diagnosis and treatment of colorectal carcinomas in the past 10 years, but some problems in detecting and managing this disease remain. A deeper understanding of the mechanism of colorectal carcinomas at the molecular level improves early diagnosis, prognostic evaluation, and disease control [2]. Recently, the relationship of anticancer effects between colorectal carcinomas and peroxisome proliferator-activated receptors (PPARs) has been reported [3, 4].

PPARs are nuclear hormone receptors and are expressed in various species including humans [5]. PPARs regulate the transcription of several genes involved in lipid metabolism, energy utilization, and storage [6], and PPARs consist of three subtypes (PPAR-*α*, PPAR-*β*/*δ*, and PPAR-*γ*) [7, 8]. PPAR-*γ* can modulate the growth and differentiation of colorectal cancer cells [9], and ligand-dependent PPAR-*γ* activation influences subcellular localization in colorectal carcinoma cell lines [10]. PPAR-*α* is predominantly expressed in tissues that catabolize high amounts of fatty acids such as the liver, kidney, and heart [11]. PPAR-*α*  regulates important cellular functions, including cell proliferation, differentiation, energy metabolism, oxidative stress, inflammation, circadian rhythms, immune response, and cell differentiation. Agonists of PPAR-*α* are widely used to treat hyperlipidemia because of its lipid metabolism promotion properties [12], and PPAR-*α* agonists also have anticancer effects [13, 14] and effects on various diseases [15–17].

Fenofibrate, a PPAR-*α*  agonist, is used to treat different forms of hyperlipidemia and regulates transcription to control lipid transportation and metabolism [18]. Fenofibrate may have anticancer effects by directly attenuating tumor growth [19–21].

PPAR-*α* is localized to the nucleus in breast carcinoma by immunohistochemistry [22]. The expression of PPAR-*α* is significantly elevated in endometrial cancer compared with that in normal endometrium [23]. However, the relationship between the expression of PPAR-*α* and clinicopathologic factors in colorectal carcinoma is unclear.

In this study, we investigated the effect of PPAR-*α* activation by fenofibrate on cell proliferation in two colorectal carcinoma cell lines. Furthermore, we examined the localization of PPAR-*α* expression in colorectal carcinoma and the correlation between PPAR-*α*  expression and clinicopathologic factors in human colorectal carcinoma tissue.

## 2. Materials and Methods

### 2.1. Cell Culture

Caco-2 cells were obtained from the American Type Culture Collection (Manassas, VA, USA), and SW620 cells were obtained from Dainippon Sumitomo Pharma (Osaka, Japan). Cells were cultured in Dulbecco's Eagle's medium (Wako Pure Chemical Industries, Osaka, Japan) supplemented with 10% heat-inactive fetal bovine serum (Biological Industries, Beit HaEmek, Israel) and 1% penicillin-streptomycin (Wako Pure Chemical Industries) at 37°C in a humidified incubator with 5% CO_2_ [24].

### 2.2. RNA Analysis

Total RNA was extracted using an RNeasy Plus Mini Kit (Qiagen, Shanghai, China) following the manufacturer's protocol. Reverse transcription polymerase chain reaction (RT-PCR) was performed using a SuperScript VILO cDNA Synthesis Kit (Invitrogen, Thermo Fisher Scientific, Waltham, MA, USA). Specific primers for PPAR-*α* were synthesized as follows: PPAR-*α* forward, 5′-CTGTCGGGATGTCACACAAC-3′ and reverse, 5′-CCGCAAACACCTACTGGATT-3′; GAPDH forward, 5′-CAACGACCACTTTGTCAAGC-3′ and reverse, 5′-TCTTCAAGGGGTCTACATGG-3′. The samples were initially denatured at 95°C for 10 min prior to the thermal cycle (PC-707, ASTEC, Fukuoka, Japan). The thermal cycler for PCR was as follows: 95°C for the 30 s, 63°C for 30 s, and 72°C for 1 min for 35 cycles. The PCR products were separated on a 2% agarose gel and visualized under LED 100 illumination (AMZ System Science, Osaka, Japan).

### 2.3. Protein Extraction and Western Blotting

Western blotting was performed as described previously [24]. Cells were seeded on 60-mm dishes and incubated at 37°C until confluent. Cells were washed with PBS, dissolved by lysis buffer (50 mM Tris-HCl, 1% Triton X-100, 150 mM NaCl, 10 mM MgCl_2_, 1 mM EDTA), and incubated on ice for 20 min. Lysates were centrifuged at 20,630 x* g* for 20 min at 4°C. The supernatants were collected, and 2× sample buffer (Nacalai Tesque, Kyoto, Japan) was added and heated for 3 min to denature the proteins. Western blotting was performed after protein separation by electrophoresis on an SDS polyacrylamide gel (Oriental Instruments, Kanagawa, Japan). Proteins separated by SDS-PAGE were transferred to a polyvinylidene fluoride (PVDF) membrane (Merck KGaA, Darmstadt, Germany) by an electrophoretic transfer system. The PVDF membrane was blocked at room temperature using 2% skimmed milk powder and then incubated with mouse anti-PPAR-*α* monoclonal antibody (1:500, Santa Cruz Biotechnology, San Francisco, CA, USA, catalog no. sc-398394), rabbit anti-*β*-actin monoclonal antibody (1:5,000, Cell Signaling Technology, Danvers, MA, USA, catalog no. 4970S), or anti-Lamin B2 antibody (1:5,000, Novus Biologicals, Littleton, CO, USA, catalog no. NBP2-43834). The membrane was washed and incubated with horseradish peroxidase-conjugated anti-rabbit antibody (1:5,000, Cell Signaling Technology, Danvers, MA, USA, catalog no. 4074S) or horseradish peroxidase-conjugated anti-mouse antibody (1:5,000, Cell Signaling Technology, Danvers, MA, USA, catalog no. 4076S) at room temperature for 1 h. Immunoreactivity was detected with an enhanced chemiluminescence kit Chemi-Lumi One L (Nacalai Tesque). Protein levels were quantified by ImageJ (version 1.52a) [25], and *β*-actin was used as an internal control.

### 2.4. Isolation of Cytoplasm and Nucleus

Cytoplasmic and nuclear proteins were isolated using a Cytoplasmic & Nuclear Protein Extraction Kit (101Bio, Palo Alto, CA, USA) according to the manufacturer's instructions. Fractions were immunoblotted with appropriate antibodies. Lamin B2 was used as a nuclear marker.

### 2.5. Cell Proliferation Analysis

Cell proliferation was measured by an MTT Cell Proliferation Assay Kit (Cayman Chemical, Ann Arbor, MI, USA) according to the manufacturer's instructions. In brief, cells were seeded onto 96-well microplates (Thermo Fisher Scientific, Waltham, MA, USA) at a density of 8 × 10^4^ cells/mL in a volume of 100 *μ*L culture media for 24 h. Cells were then incubated in culture medium with fenofibrate, or fenofibrate and GW6471 for 48 h. MTT reagent was added in each well and incubated at 37°C for 3 h. Afterwards, Crystal Dissolving Solution (Cayman Chemical) was added into each well and further incubated at 37°C for 6 h. The optical density values (OD value) were measured at 570 nm using a model 680 Microplate Reader (Bio-Rad Laboratories, Hercules, CA, USA).

### 2.6. Hoechst Staining

Apoptosis was detected by Hoechst staining for analysis of chromatin condensation or nuclear fragmentation. In brief, cells were seeded onto 60-mm dishes at a density of 1 × 10^5^ cells/mL in a volume of 4 mL culture media for 24 h. Cells were incubated in culture medium with fenofibrate, or fenofibrate and GW6471 for 48 h. The cells were harvested and stained by 1 mg/mL bisbenzimide H 33258 (Sigma, St. Louis, MO, USA). The nucleus of the stained cells was observed in several fields by a fluorescence microscope (Nikon Eclipse E600, Nikon Instruments, Melville, NY, USA), and the ratio of apoptotic cells was determined.

### 2.7. Immunocytochemistry

The cells were seeded on Nunc Lab-Tek chamber slides (Thermo Fisher Scientific) and incubated at 37°C until confluent. Cells were subsequently washed twice with PBS and fixed with 3.7% formalin at room temperature for 15 min. The cells were permeabilized with 0.25% triton in PBS for 10 min and blocked with 2% bovine serum albumin (Wako Pure Chemical Industries) in PBS for 1 h. Cells were incubated at room temperature for 2 h with HRP-labeled anti-PPAR-*α* antibody (1:200, Santa Cruz Biotechnology, catalog no. sc-398394). The cells were rinsed three times with PBS and then stained with 3,3′-diaminobenzidine tetrahydrochloride (DAB) using a DAB substrate kit (Nichirei Biosciences, Tokyo, Japan). The cells were counterstained with Meyer's hematoxylin, dehydrated, cleared with 99% xylene for 15 min, and mounted in malinol (Muto Pure Chemicals, Tokyo, Japan). The expression of PPAR-*α* in cells was observed at 200x magnification using a light microscope (BX53; Olympus Corporation, Tokyo, Japan), and images were captured with a microscopic camera (DP20-5; Olympus Corporation).

### 2.8. Subjects

Tissues were obtained from 64 cases diagnosed as colorectal carcinoma and surgically resected between April 2012 and March 2014 at Kagawa University Hospital (Kagawa, Japan). Clinicopathological factors were classified according to histological differentiation degree, lymphatic invasion, venous invasion, lymph node metastasis, depth of invasion, and stage according to the Japanese Classification of Colorectal Carcinoma (8th Edition). All subjects provided written informed consent. The study was conducted with the approval of the Institutional Research Ethics Committee of Kagawa Prefectural University of Health Sciences (Kagawa, Japan).

### 2.9. Immunohistochemistry

Immunohistochemistry was performed as described previously [26]. The tissue sections were incubated at room temperature for 2 h with the following primary antibody: HRP-labeled anti-PPAR-*α* antibody (1:200, Santa Cruz Biotechnology, catalog no. sc-398394). Slides were rinsed three times with PBS and then stained with DAB using the DAB substrate kit (Nichirei Biosciences). Sections were counterstained with Meyer's hematoxylin, dehydrated, cleared with 99% xylene, and then mounted in malinol. The expression of PPAR-*α* in cells was observed at 200x magnification using a light microscope (BX53; Olympus Corporation, Tokyo, Japan), and images were captured with a microscopic camera (DP20-5; Olympus Corporation). The classification of nuclear PPAR-*α* expression was assessed using the following scoring: no staining, 0; <25% positive cells, 1+; 25-50% positive cells, 2+; 50-75% positive cells, 3+; and >75% positive cells, 4+. The expression levels of PPAR-*α*  were grouped into negative (0, 1+, and 2+) and positive (3+ and 4+) groups.

### 2.10. Statistical Analysis

Protein expression by western blotting, the survival rate of colorectal carcinoma cell by MTT assay, and the proportion of cells producing apoptosis by Hoechst stain were analyzed using a paired Student's t-test. The correlation between immunohistochemical staining and clinicopathological factors was examined using the *χ*^2^ test. P<0.05 was considered significant. All statistical analyses were performed using SPSS 24.0 software (IBM SPSS, Armonk, NY, USA).

## 3. Results

### 3.1. Expression of PPAR-*α* in Colorectal Carcinoma Cell Lines

**Figure 1:**
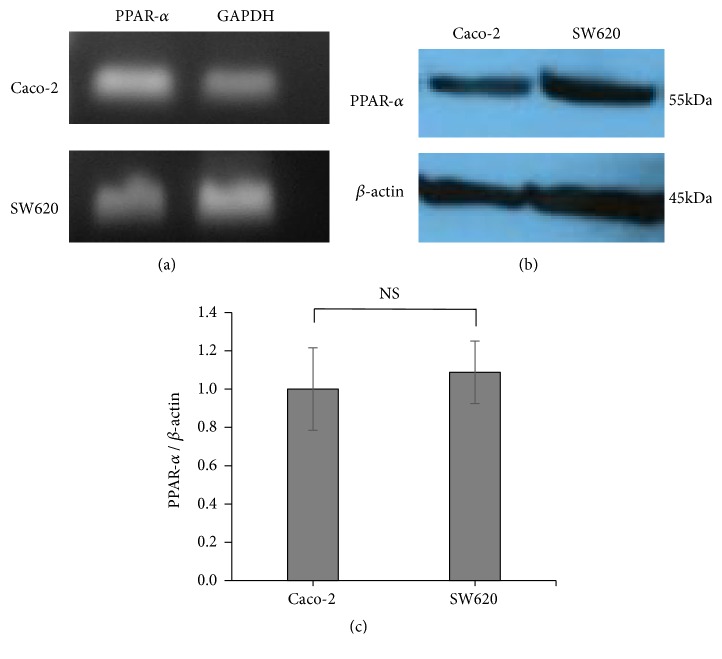
PPAR-*α* mRNA and protein are expressed in colorectal carcinoma cells. (a) RT-PCR analysis of PPAR-*α* in Caco-2 and SW620 cells. (b) PPAR-*α* protein was assayed by western blot in Caco-2 and SW620 cells. (c) PPAR-*α* protein levels were calculated and compared with those of Caco-2 and SW620 cells. Error bars indicate standard deviation.

Expression of PPAR-*α* mRNA and PPAR-*α* protein levels in colorectal carcinoma cell lines (Caco-2 and SW620 cells) was examined. First, expression of PPAR-*α*  mRNA was examined by RT-PCR in a colorectal carcinoma cell line. We confirmed the expression of PPAR-*α* RNA in Caco-2 and SW620 cells (Figure 1(a)). In addition, expression of PPAR-*α*  protein was examined by western blotting in the colorectal carcinoma cell lines, and there was no difference in the amount of PPAR-*α*  protein in Caco-2 and SW620 cells (Figures 1(b) and 1(c)).

### 3.2. Localization of PPAR-*α*  in Caco-2 and SW620 Cells

**Figure 2:**
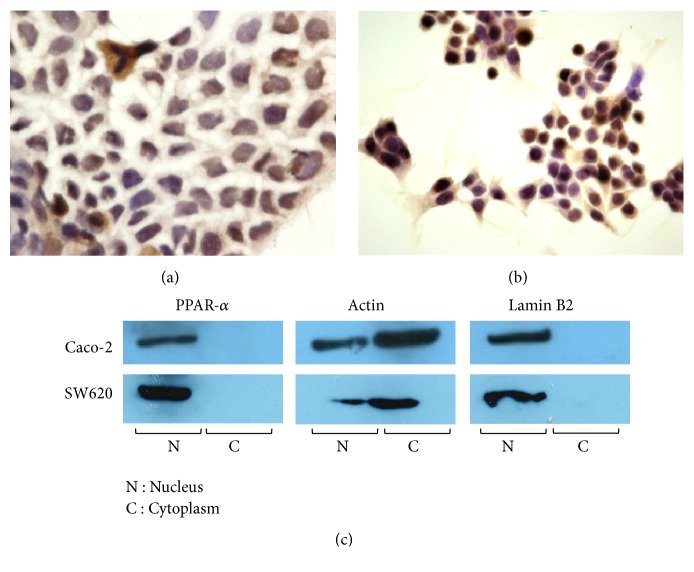
Localization of PPAR-*α* in Caco-2 and SW620 cells. (a, b) Immunocytochemical staining of PPAR-*α* in Caco-2 and SW620 cells. (c) PPAR-*α* was expressed in the nucleus of Caco-2 and SW620 cells.

Immunocytochemical analysis was performed to determine the subcellular localization of PPAR-*α* in Caco-2 and SW620 cells. PPAR-*α*  was mainly localized in the nucleus of the colorectal carcinoma cell lines (Figures 2(a) and 2(b)). Next, Caco-2 and SW620 cells were divided into cell fractions (nucleus and cytoplasm), and the expression of PPAR-*α*  was confirmed by comparing the amount of PPAR-*α*  protein by western blotting. PPAR-*α*  was expressed in the nucleus and was not expressed in the cytoplasm (Figure 2(c)).

### 3.3. Fenofibrate Inhibits the Viability of Colorectal Carcinoma Cells by MTT Assay

**Figure 3:**
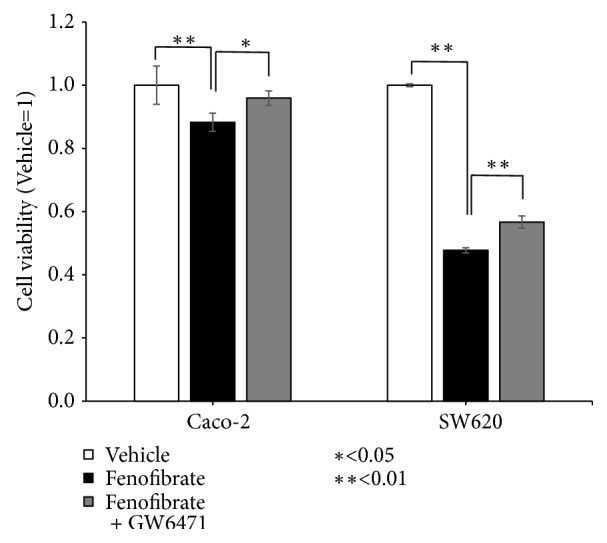
PPAR-*α* activation affects cell proliferation. Human colorectal carcinoma cells (Caco-2 and SW620) were treated with fenofibrate (200 *μ*M for Caco-2 cells and 100 *μ*M for SW620 cells) and 3 *μ*M of GW6471 for 48 h, and cell proliferation was determined by an MTT assay. Error bars indicate standard deviation.

The effect of different concentrations of fenofibrate on the viability of colorectal carcinoma cells was evaluated using an MTT assay. Colorectal carcinoma cells were treated with fenofibrate (200 *μ*M for Caco-2 cells and 100 *μ*M for SW620 cells; DMSO was used as a control for each group) and incubated for 48 h (Figure 3). Fenofibrate significantly decreased Caco-2 and SW620 cell viability (P<0.01 and P<0.01, respectively). GW6471 (3 *μ*M), an antagonist of PPAR-*α*, was added to fenofibrate (200 *μ*M for Caco-2 cells and 100 *μ*M for SW620 cells), which significantly suppressed the antiproliferative effect of PPAR-*α*  by fenofibrate in Caco-2 and SW620 cells (P<0.05 and P<0.01, respectively) (Figure 3).

### 3.4. Fenofibrate Increases Apoptotic Cells

**Figure 4:**
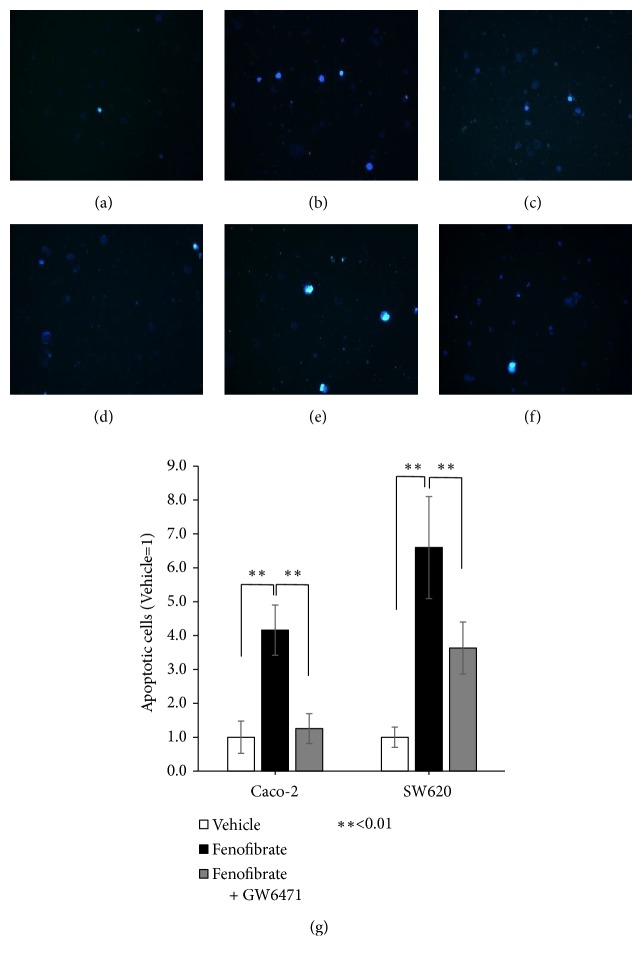
PPAR-*α* activation affects apoptosis. (a) Control in Caco-2 cells (200×). (b) 100 *μ*M of fenofibrate in Caco-2 cells (200×). (c) 100 *μ*M of fenofibrate and 3 *μ*M of GW6471 in Caco-2 cells (200×). (d) Control in SW620 cells (200×). (e) 100 *μ*M of fenofibrate in SW620 cells (200×). (f) 100 *μ*M of fenofibrate and 3 *μ*M of GW6471 in SW620 cells (200×). (g) Human colorectal carcinoma cells (Caco-2 and SW620) were treated with 100 *μ*M of fenofibrate, and 100 *μ*M of fenofibrate and 3 *μ*M of GW6471 for 48 h; apoptosis was determined by a Hoechst stain. Error bars indicate standard deviation.

A Hoechst stain was used to examine apoptotic Caco-2 and SW620 cells after treatment with fenofibrate (100 *μ*M for Caco-2 and SW620 cells; DMSO was used as a control for each group). The number of apoptotic cells was significantly increased by addition of fenofibrate to Caco-2 and SW620 cells (P<0.01 and P<0.01, respectively) (Figure 4). GW6471 (3 *μ*M) was added to fenofibrate (100 *μ*M), which significantly suppressed the increase of Caco-2 and SW620 apoptotic cells (P<0.01 and P<0.01, respectively) (Figure 4).

### 3.5. Relationship between PPAR-*α*  Expression and Clinicopathological Factors by Tissue Immunohistochemistry

**Figure 5:**
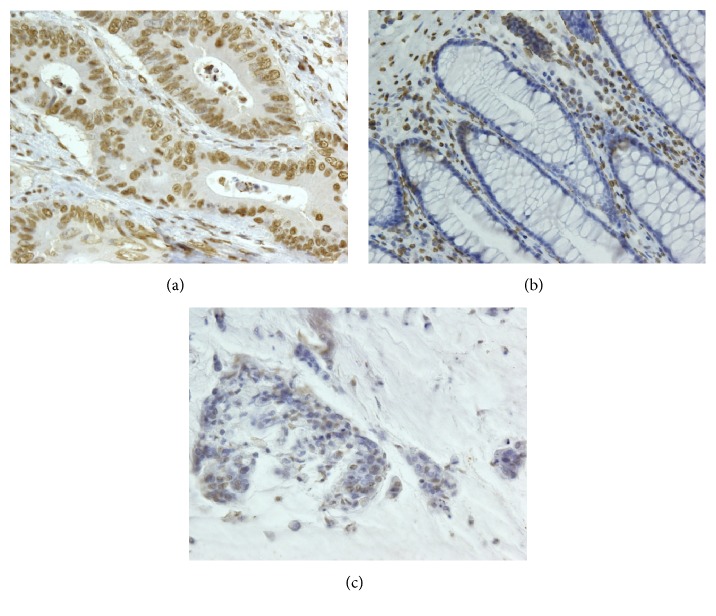
Expression of PPAR-*α* in different histological types. (a) Positive PPAR-*α* expression in well-to-moderately differentiated adenocarcinoma (200×). (b) Negative PPAR-*α* expression in normal epithelium tissue (200×). (c) Negative PPAR-*α* expression in mucinous adenocarcinoma (200×).

Paraffin-embedded colorectal carcinoma tissue blocks were provided by Kagawa University Hospital (Kagawa, Japan). There were 64 patients (39 males and 25 females) with a mean age of 68.6 ± 12.6. Fifty-two cases were differentiated carcinoma types, and 12 cases were mucinous adenocarcinoma. Forty-eight cases were positive and 16 cases were negative for lymphatic invasion; 48 cases were positive and 16 cases were negative for venous invasion. Lymph node metastasis was observed in 21 cases. For the depth of invasion, 1 case was T0, 5 cases were T1, 3 cases were T2, 34 cases were T3, and 21 cases were T4. For the progress (stage) classification, 1 case was stage 0, 7 cases were stage I, 34 cases were stage II, 21 cases were stage III, and 1 case was stage IV. PPAR-*α*  expression was mainly localized in the nucleus in well-to-moderately differentiated cancer cells (Figure 5(a)), and almost no expression was observed in normal epithelium tissue (Figure 5(b)). PPAR-*α*  expression increased in colorectal carcinoma tissue and decreased in normal epithelium tissue (P<0.01) (Table 1). As shown in Table 2, PPAR-*α*  expression was not related to sex, age, lymphatic invasion, venous invasion, lymph node metastasis, depth of invasion, and stage. However, the expression of PPAR-*α* declined in mucinous adenocarcinoma (Figure 5(c)), and PPAR-*α* expression in well-to-moderately differentiated adenocarcinoma was significantly higher than in mucinous adenocarcinoma (P<0.05) (Table 2).

## 4. Discussion

We examined the activity of PPAR-*α* by fenofibrate in colorectal carcinoma cell lines. PPAR-*α* was involved in cell survival because fenofibrate decreased cancer cell proliferation and induced apoptosis, which was suppressed by addition of GW6471 in colorectal carcinoma cells (Figures 3 and 4(g)). PPAR-*α*-dependent mechanisms have been used to explain the anticancer effects of fenofibrate in breast cancer [27], pancreatic cancer [28], ovarian cancer [29], endometrial cancer [30], neuroblastoma cells [31], proximal tubular cells [32], lymphoma, and multiple myeloma [33], but little has been reported about PPAR-*α* in colorectal carcinoma. Fenofibrate decreased cell proliferation and increased apoptosis of colorectal carcinoma cells, which expressed PPAR-*α* (Figures 2(a) and 2(b)). These findings indicated that PPAR-*α* has possible anticancer effects in colorectal carcinoma.

PPAR-*α* was expressed in the nucleus of carcinoma cells (Figures 2(a), 2(b), and 2(c)). PPAR-*γ* is expressed in the nucleus of cancer cells in breast cancer [22], but PPAR-*γ* is expressed in the cytoplasm and nucleus in renal cell carcinoma [34]. However, the localization of PPAR-*α* in colorectal carcinoma tissue has not been reported. The expression of nuclear PPAR-*α* is significantly increased in endometrial cancer compared with that of normal endometrium [23]. Additionally, neoplastic cells have strong PPAR-*α* expression and activity, which increase metabolic rates in cancer cells [23]. We found that PPAR-*α* was expressed in the nucleus in large intestine carcinogenetic tissue.

We found that PPAR-*α* expression in normal epithelium tissue was lower than in well-to-moderately differentiated adenocarcinoma (Figures 5(a) and 5(b)) (Table 1). Moreover, we examined the correlation between clinicopathological factors and PPAR-*α* expression and localization in colorectal carcinoma. PPAR-*α* expression in well-to-moderately differentiated adenocarcinoma was significantly higher than in mucinous adenocarcinoma (Figures 5(a) and 5(c)) (Table 2). Colorectal mucinous adenocarcinoma accounts for 5-20% of colorectal carcinoma and is characterized by viscous liquid (>50% of the tumor region) in the extracellular space [35, 36]. There are no reports of PPARs in mucinous adenocarcinoma. The cultured cells in this study were established from primary colorectal carcinoma patients (Caco-2) and metastasized lymph nodes (SW620), and these cells do not produce mucus. PPAR-*α* was expressed in the nucleus of colorectal carcinoma cells, which do not produce mucus in vivo and in vitro (Figures 2(a), 2(b), and 5(a)). Compared with well-to-moderately differentiated adenocarcinoma, mucinous adenocarcinoma has a larger tumor size and higher pathological stage, and patients with mucinous adenocarcinoma have an increased risk of metastasis and worse prognosis [37].

PPAR-*α* was localized in the nucleus of colorectal carcinoma (Figures 5(a) and 5(b)), and fenofibrate induced apoptosis and suppressed tumor proliferation by activating PPAR-*α* in cultured cells (Figures 3 and 4). These findings suggest that activation of PPAR-*α* by fenofibrate may inhibit colorectal carcinoma cell survival. PPARs are nuclear receptors that are activated in the nucleus to regulate various transcription factors. PPAR-*γ* ligands were previously shown to significantly induce apoptosis in colon cancer cells by suppressing the activity of NF-*κ*B, which is a transcription factor of Bcl-2, and inhibiting the expression of antiapoptotic Bcl-2 proteins [38–40]. NF-*κ*B and Bcl-2 have been suggested to play important roles in the activation of PPAR-*γ* and apoptosis in human colon cancer cells [39]. Previous studies reported the downregulation of NF-*κ*B signaling in fenofibrate-related apoptosis [27, 41, 42]. A PPAR-*α* ligand was shown to induce apoptosis in breast cancer cells, which may have been due to the inhibition of NF-*κ*B [27], and the activation of PPAR-*α* also decreased the growth rate of breast cancer cells by reducing the levels of various cell cycle-regulating cyclins [43]. Therefore, Bcl-2 and its transcription factor, NF-*κ*B, may suppress tumor proliferation via the activation of PPAR-*α* in colorectal carcinoma. However, the mechanisms underlying the relationship between tumor cell proliferation, including apoptosis, and the transcriptional activity of PPAR-*α* remain unclear. Further studies are needed to clarify the mechanisms by which PPAR-*α* regulates the induction of apoptosis in colorectal carcinoma.

In summary, we found that PPAR-*α*  activation by fenofibrate reduced the survival rate of colorectal carcinoma cells. Moreover, PPAR-*α*  expression increased in colorectal carcinoma compared with that in normal epithelium tissue. PPAR-*α* activation by agonists may have an anticancer effect on colorectal adenocarcinoma, and nuclear expression of PPAR-*α* may be a therapeutic target for colorectal adenocarcinoma.

## Figures and Tables

**Table 1:** PPAR-*α* expression in the normal epithelium tissues (n=37) and colorectal carcinoma tissues (n=64).

	PPAR-*α*		P-value
	(-)	(+)	
Normal epithelium tissue (n=37)	35	2	<0.01
Carcinoma tissue (n=64)	14	50	

**Table 2:** The relationship of PPAR-*α* expression to clinicopathologic parameters of colorectal carcinoma (n=64).

Parameters		Number of	PPAR-*α*		P-value
		cases	(-)	(+)	
Sex					
	Male	39	8	31	0.985
	Female	25	6	19	
Age					
	≤65	31	8	23	0.664
	>65	33	6	27	
Histological type					
	Well-to-moderately differentiated	52	8	44	0.026
	Mucinous	12	6	6	
Lymphatic invasion					
	Positive	48	11	37	1.000
	Negative	16	3	13	
Venous invasion					
	Positive	48	10	38	1.000
	Negative	16	4	12	
Lymph node metastasis					
	Positive	21	5	16	1.000
	Negative	43	9	34	
Depth of invasion					
	T0	1	0	1	0.734
	T1	5	0	5	
	T2	3	1	2	
	T3	34	8	26	
	T4	21	5	16	
Stage					
	0	1	0	1	0.970
	I	7	1	6	
	II	34	8	26	
	IIIa	17	4	13	
	IIIb	4	1	3	
	IV	1	0	1	

## Data Availability

The data used to support the findings of this study are available from the corresponding author upon request.
